# Prediction of smoking by multiplex bisulfite PCR with long amplicons considering allele-specific effects on DNA methylation

**DOI:** 10.1186/s13148-018-0565-1

**Published:** 2018-10-23

**Authors:** Nikolay Kondratyev, Arkady Golov, Margarita Alfimova, Tatiana Lezheiko, Vera Golimbet

**Affiliations:** Clinical Genetics Laboratory, Mental Health Research Center, Moscow, Russia

**Keywords:** Allele-specific methylation, Clinical sequencing, DNA methylation, Single-molecule sequencing, Smoking, Schizophrenia, Targeted sequencing

## Abstract

**Background:**

Methylation of DNA is associated with a variety of biological processes. With whole-genome studies of DNA methylation, it became possible to determine a set of genomic sites where DNA methylation is associated with a specific phenotype. A method is needed that allows detailed follow-up studies of the sites, including taking into account genetic information. Bisulfite PCR is a natural choice for this kind of task, but multiplexing is one of the most important problems impeding its implementation. To address this task, we took advantage of a recently published method based on Pacbio sequencing of long bisulfite PCR products (single-molecule real-time bisulfite sequencing, SMRT-BS) and tested the validity of the improved methodology with a smoking phenotype.

**Results:**

Herein, we describe the “panhandle” modification of the method, which permits a more robust PCR with multiple targets. We applied this technique to determine smoking by DNA methylation in 71 healthy people and 83 schizophrenia patients (*n* = 50 smokers and *n* = 104 non-smokers, Russians of the Moscow region). We used five targets known to be influenced by smoking (regions of genes *AHRR*, *ALPPL2*, *IER3*, *GNG12*, and *GFI1*). We discovered significant allele-specific methylation effects in the *AHRR* and *IER3* regions and assessed how this information could be exploited to improve the prediction of smoking based on the collected DNA methylation data. We found no significant difference in the methylation profiles of selected targets in relation to schizophrenia suggesting that smoking affects methylation at the studied genomic sites in healthy people and schizophrenia patients in a similar way.

**Conclusions:**

We determined that SMRT-BS with “panhandle” modification performs well in the described setting. Additional information regarding methylation and allele-specific effects could improve the predictive accuracy of DNA methylation-based models, which could be valuable for both basic research and clinical applications.

**Electronic supplementary material:**

The online version of this article (10.1186/s13148-018-0565-1) contains supplementary material, which is available to authorized users.

## Background

Whole-genomic DNA methylation studies of different human phenotypes based on DNA microarrays are the most common and cost-effective variant of epigenome-wide association studies (EWAS). The goal of such studies is to define DNA methylation features of a particular phenotype. This knowledge could be exploited further to understand the biology of the trait and ultimately to make predictions about phenotype by means of DNA methylation. There is a range of phenotypes explored with EWAS, like, for instance, autoimmune diseases [1–3], cancers [4–6] and psychiatric conditions [7–11]. However, not all of EWAS has resulted in the discovery of well-reproduced sets of genomic targets. The examples of such successful EWAS are those studies of ageing [12, 13], obesity [14], alcohol intake [15] and smoking [16].

There are many reasons why EWAS may fail. Undersampling, poor choice of cellular model, cell-type heterogeneity, unbalanced confounders and genetic effects could be the main issues [17, 18]. Genetic factors are of particular importance because allele-specific DNA methylation (ASM) effects are known to affect around 10% of CpGs and account for a significant portion of DNA methylation variability [19–22]. The ubiquitous character of ASM means that a DNA methylation study is supposed to include genetic factors in its experimental design. ASM impacts could be an independent subject of research. For example, there is an expectation that certain unexplained genetic effects, produced en masse by genome-wide association studies (GWAS), could be explained by ASM [22, 23].

If the methylation targets for a particular trait are already established, it is possible to employ them as biomarkers in clinical or forensic applications. The BLUEPRINT consortium has recently evaluated the possibility of utilising DNA methylation biomarkers in clinical practice through a large multicentre study and found that the technology is ready for application in practical terms. It has been concluded that targeted bisulfite PCR-based methods followed by next-generation sequencing (NGS) outperform or are on par with alternatives in terms of accuracy and robustness [24].

The existing EWAS-based DNA methylation biomarkers are essentially sets of multiple genomic targets where DNA methylation is linked to a specific phenotype. The number of targets in a single set varies for a particular phenotype, but usually, it is quite large. The accuracy of predictions based on less or more expansive sets of targets is explored, for example, with DNA methylation-based age prediction, with evidence strongly favouring the latter [25]. The reasonable assumption is that in the future, more powerful EWAS will provide more signals for more accurate prediction. This creates a demand for very multiplex yet targeted approaches for the detection of DNA methylation.

Yang et al. reported that relatively long PCR products (up to 2 Kbp) could be routinely amplified with genomic DNA, which was bisulfite converted with certain specific commercial kits [26]. As the conventional Illumina sequencing is not able to read through such long amplicons, the Pacbio sequencing platform was used [27]. The method, dubbed “single-molecule real-time bisulfite sequencing” (SMRT-BS), is especially suitable for an allele-specific methylation assessment. The longer reads provide more detailed data for the estimation of local methylation signal and are more likely to capture local genetic context.

In the present study, we addressed the question of how this additional methylation and genetic information could enhance the accuracy of DNA methylation-based biomarkers on a well-established set of smoking EWAS hits [28, 29]. The PCR with bisulfite-converted DNA (bisulfite PCR) is considered to be more difficult than conventional PCR. This is often explained by the partial degradation of DNA during the conversion [30]. In addition, the converted DNA is basically in a three-letter code (save the unconverted cytosines), which makes it harder to produce a specific PCR product. Longer amplicons are even more difficult to harvest because longer fragments are less represented in converted DNA samples and the converted DNA is a harder template being AT-rich and containing uracils and cytosine-5-methylenesulfonates instead of unmethylated and 5-hydroxymethylated cytosines, respectively [31]. Though these problems for various amplicons can be circumvented with careful primer design and optimization of PCR parameters, it seems like an insurmountable obstacle for the multiplex bisulfite PCR, especially for the longer PCR products. We made use of a modified (“panhandle”) SMRT-BS method with the objective of resolving those problems, making it more robust and multiplex-friendly.

We validated this approach by studying the interaction between smoking and schizophrenia. It has been shown that smoking is a major covariate that needs to be controlled for in schizophrenia EWAS. In particular, genomic signals within the regions of *AHRR*, *IER3*, and *GFI1* genes have been found in raw uncontrolled EWAS [32]. It is possible that the smoking exposure manifests itself differently in smoking-related targets of patients with schizophrenia. We applied the “panhandle” SMRT-BS method to assess whether methylation in smoking-associated regions depends on the disease.

## Methods

### Sample

Participants were selected from a database of the Mental Health Research Center (MHRC) in Moscow. There were 83 schizophrenia patients from the MHRC or Moscow Psychiatric Hospital No. 1 and 71 healthy controls. All the participants provided written informed consent and donated blood samples for DNA extraction. Smoking was assessed through oral interviews, and the smoking status of patients was double-checked with their psychiatrists. Current smokers and never smokers, hereinafter referred to as smokers and non-smokers, respectively, participated in this study. The sample consisted of 50 smokers (mean age 28.0 ± 7.5 years, 40% women, 54% patients) and 104 non-smokers (mean age 26.0 ± 5.9 years, 54% women, 54% patients).

### DNA extraction and bisulfite conversion

Genomic DNA was extracted with the DNeasy Blood and Tissue Kit (Qiagen, USA) according to the manufacturer’s instructions. The bisulfite-converted DNA samples were obtained with the EpiGentek Methylamp DNA Modification Kit (Epigentek Group Inc., USA) in agreement with the manufacturer’s protocol. We did support the original Yang et al. [26] conclusion that this particular kit worked better with the long bisulfite PCR compared to the Epitect Fast DNA Bisulfite Kit (Qiagen, USA).

### Bisulfite primer design

Primers were designed with the primer3 software [33] to amplify approximately 1.3 Kbp PCR products of converted genome sequences. Primers were designed to be of 25–35 bp length, Tm = 60 °C and no CpGs allowed. The designed primer sequences are listed in the Additional file 1: Table S1. The summary information surrounding the amplicons is found in Table 1.

**Table 1:** Amplicons utilised in the study

Reference (index) CpG, Illumina ID	The closest gene to the reference CpG	Genome coordinate (hg19) of the amplicon	DNA strand of the amplicon	Length of the amplicon, bp	Amount of CpGs in the amplicon	Reference
cg05575921	*AHRR*	chr5:372478-373819	+	1342	44	[28, 29, 63–70]
cg21566642	*ALPPL2*	chr2:233283630-233284930	−	1301	114	[28, 29, 63–66, 68, 70]
cg06126421	*IER3*	chr6:30719327-30720645	+	1319	19	[28, 29, 65, 66, 68, 70]
cg25189904	*GNG12*	chr1:68298855-68300158	−	1304	85	[28, 29, 64, 68, 69]
cg09935388	*GFI1*	chr1:92947265-92948622	+	1358	73	[28, 29, 67, 68, 70]
cg15417641	*CACNA1D*	chr3:53699512-53700811	−	1300	17	[28, 29, 68]

### Bisulfite PCR

For the bisulfite PCR, we utilised 20 ng of the converted DNA, 1 μM of the “panhandle” 5′-phosphorylated primer “U1” GCAGTCGAACATGTAGCTGACTCAGGTCAC, 5 nM of each of the specific primer with the identical U1 sequence on the 5′ end and 200 nM dNTP, 1 mg/ml BSA, 2.5 U HotTaq polymerase with the corresponding buffer (Sileks, Russia) in a total volume of 12.5 μl. The choice of polymerase is important—the polymerase should be a simple hot-start polymerase that, unlike specialised high-fidelity polymerases, is not capable of overcoming the suppression effect. We have routinely verified the PCR kinetics with the 20× EVA Green DNA intercalating dye (Biotium Inc., USA), which apparently did not affect the reaction. The PCR programme was as follows: (1) initial denaturation, 94 °C, 10 min; (2) 5 cycles of specific PCR (94 °C, 20 s; 55 °C, 1 min; 64 °C, 4 min); (3) 37 cycles of “panhandle” PCR (94 °C, 20 s; 64 °C, 2 min); and (4) final incubation, 64 °C, 10 min.

### Barcoding

For the creation of the Y-adapters, we employed 96 unique combinations of two sets of oligonucleotides: a first set of eight oligonucleotides CGAGTAGTGTTC-unique 5-letter sequence-CAAGGCACACAGGGGATAGG and a second set of 12 oligonucleotides 5′-CCATCTCATCCCTGCGTGTC-unique five-letter sequence-CTACACTACTCGT. A combination of two oligonucleotides from both sets could be used to create 96 unique Y-adapters. The oligonucleotides from the first set were 5′-phosphorylated. The sequences of oligonucleotides bearing molecular barcodes are found in Additional file 1: Table S2. Each Y-adapter was formed by pairing of 10 nM of a single oligonucleotide from each of these two sets in an annealing reaction within 25 μl of the annealing buffer AB (10 mM Tris-HCl (pH 8.0), 50mМ NaCl, 0.1 mM EDTA). The annealing reactions were set in the PCR thermal cycler with the following programme: incubation 98 °C, 1 min; cooling down to 70 °C (1.6 °C/s); and cooling down to 10 °C (0.1 °C/s). The reactions were then diluted fivefold with AB, stored at − 20 °C and utilised as a stock solution. Immediately prior to ligation, these stocks were diluted 10-fold with 1× T4 ligase buffer with 5% PEG 4000. The ligation reactions were set in 10 μl: 2 μl diluted Y-adapters stock solution, 2 μl of PCR products and 6 μl of the ligation master mix (1.33× T4 ligase buffer with 6.67% PEG 4000, 1.2 w.u. T4 DNA ligase, Thermo, USA). The ligation reactions were performed at 20 °C for 2.5 h followed by incubation at 65 °C for 10 min. Then, the reactions were mixed in libraries (two libraries, up to 96 samples per library). The libraries (500 μl) were washed twice with 10 mM Tris-HCl (pH 8.0) and concentrated down to 50 μl by Amicon Ultra-0.5 30K Device columns (Merck, USA). Next, the libraries were washed twice to eliminate the primers, unligated Y-adapters, etc., with 0.7 volume of AMPure XP magnet beads (Beckman Coulter Inc., USA). The purified DNA solution was employed for amplification of the libraries with additional PCR. The PCR was performed with 250 nM primers, specific to the end of the Y-adapters: “emPCR_A” 5′-CCATCTCATCCCTGCGTGTC and “emPCR_B” 5′-CCTATCCCCTGTGTGCCTTG with the HiFi HotStart Uracil+ 2× master mix (Kapa Biosystems, Republic of South Africa). The PCR was performed with the following programme: initial denaturation 95 °C, 5 min; 20 cycles: 98 °C, 20 s; 60 °C, 15 s; and 72 °C, 2 min. The PCR product was then length-selected through agarose electrophoresis and purified with the QIAquick Gel Extraction Kit (Qiagen, USA).

### CCS library preparation and sequencing

The CCS library preparation (ligation of “SMRTBell” adapters with SMRTbell Template Prep Kit, Pacbio, USA) and sequencing was performed with Pacbio RSII (P6/C4 chemistry) in the facility of the Washington University Pacbio Sequencing Services. The final volume of raw data used in this paper is approximately equal to a single SMRT cell of the Pacbio RSII device.

### Post-sequencing data preparation

Only reads with a quality score of no less than Q30 (average quality score was Q40) were utilised in the following analysis. After adapter trimming, we obtained 56,581 reads with correct adapters and primer sequences. The reads were demultiplexed with no errors in the barcode sequences allowed, discarding 11% of the reads. The median amount of reads per barcode was 202 (Q1:128, Q3:315). Adapter trimming and barcode demultiplexing were performed with the cutadapt programme [34]. The alignment of the filtered reads to the reference human genome (hg19) was obtained using the bismark programme with 88% mapping efficiency [35] (together with bowtie2 [36]). Filtration of under-converted DNA (threshold of unconverted CpH < 5%, H = A/C/T) and de-duplication were performed with the perl script (see Additional file 1: Supplementary Note 2 on de-duplication procedure). The final conversion rate was no less than 0.98 for each of the analysed targets. The number of reads for each target with the different stages of data preparation is presented in Additional file 1: Table S5.

### ASM data

Each read in the data (files in SAM format) was sorted with perl script based on CIGAR string parsing by alleles of easily identifiable polymorphisms in each target. The list of used polymorphisms is presented in Additional file 1: Table S3. The rate of methylation of individual CpGs per haplotype for each sample was defined by the bismark software. Only samples with the minimum 5× read depth per haplotype were used, leading to discarding of the *CACNA1D* target owing to insufficient amounts of data. Missing values were mean imputed. Methylation signals in sites of known CpG-SNPs were not employed in the following analysis. The methylation rate for each of the CpGs was logit-transformed according to the equation: $M=\log \left({m}^{\hbox{'}}/1-{m}^{\hbox{'}}\right)$; ${m}^{\hbox{'}}=\left(m\left(n-1\right)+0.5\right)/n$, where *m* is raw methylation rate, *n* is the sample size.

### Statistical analysis

Three smoking status predictive models were tested on the prepared ASM dataset, hereinafter referred as “index”, “boruta” and “boruta.adjusted”. Age, gender and diagnosis were regressed out for subsequent analysis. The regression residuals were employed for subsequent analysis. For the “boruta.adjusted” model, the haplotype information was also used. The Boruta algorithm was used to determine important CpGs (“important” in the sense of the Boruta algorithm) inside each of the targets for the “boruta” and “boruta.adjusted” models. Original CpGs from smoking EWAS were selected for the “index” model (Table 1). The dataset was randomly split 1:1 into a train and test sets. The logistic model with selected CpGs was trained on the train set. The combined prediction logistic model was built on top of prediction values of individual target models. In the case of heterozygous samples, the prediction values were averaged. The performance of the combined models was evaluated on the test set. The analysis was conducted with the R statistical software programme with the “Boruta” package [37].

## Results

**Fig. 1:**
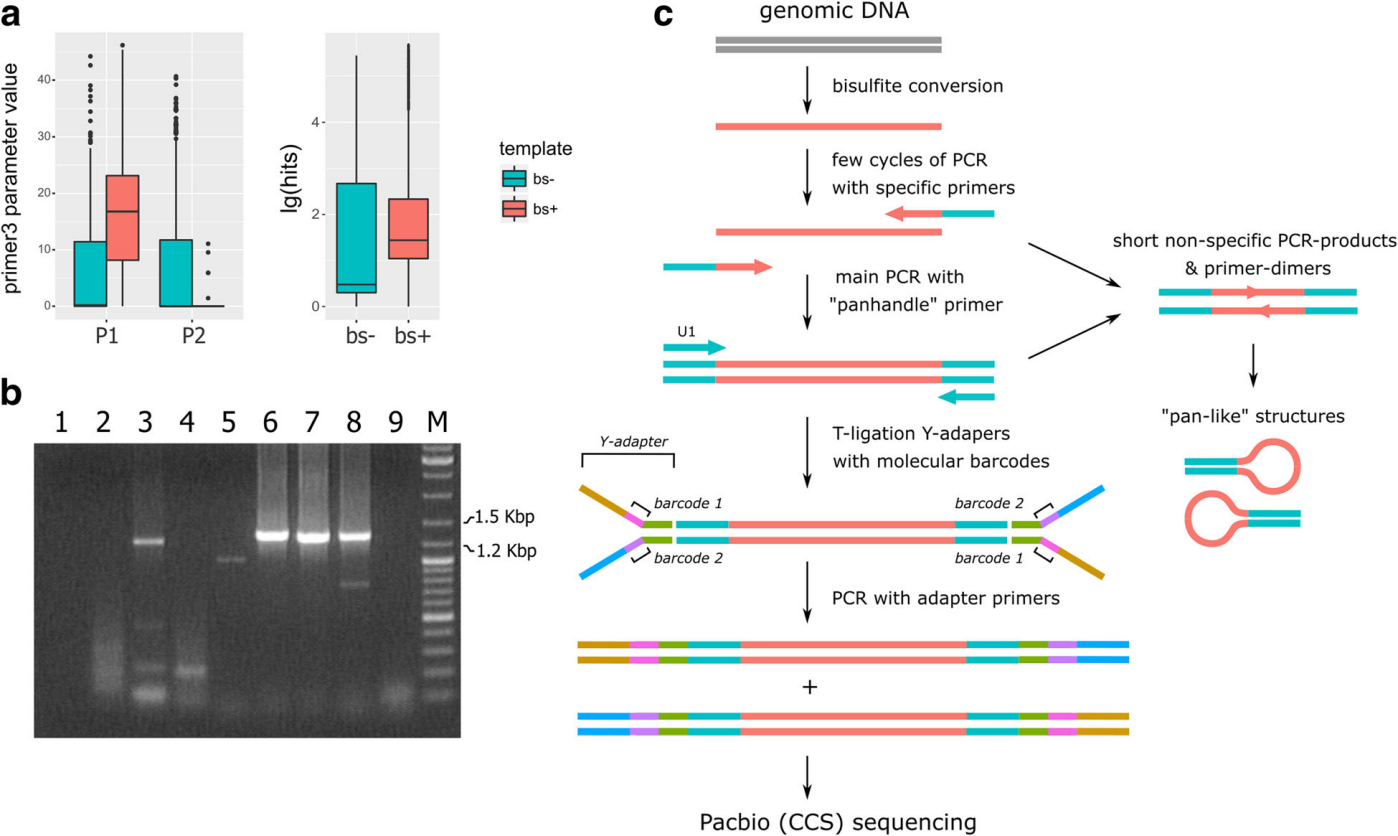
“Panhandle” SMRT-BS. **a** Computer simulation of primer performance in conventional and bisulfite PCR. The left panel shows the ability of primers to form primer-dimers with P1 = PAIR_ANY_TH and P2 = SELF_ANY_TH parameters of the primer3 output. The right panel demonstrates a tendency of the non-specific annealing by the log_10_ number of BLAST hits at 3′-ends of the primers. Further details are available in Additional file 1: Supplementary Note 1. **b** Photography of agarose gel electrophoresis of different long bisulfite PCR products. The lanes are numbered as follows: 1–4 —conventional bisulfite PCR (with specific primers without the “panhandle”), 5–9—“panhandle” bisulfite PCR with the “panhandle” U1 primer as described in the “Methods” section; 1–8 with bisulfite-converted DNA, 9—without the template; 1, 5—without specific primers; 2, 6—the AHRR target; 3, 7—the ALPPL2 target; 4, 8—six-target multiplex PCR. M—DNA ladder marker. **c** Scheme of the “panhandle” SMRT-BS approach

Our computer simulations demonstrated that typical oligonucleotides for bisulfite PCR tend to form approximately three times tighter primer-dimers and are 14 times more likely to anneal to non-specific genomic sites than primers for conventional PCR (Fig. 1a). To resolve these problems, we used a suppressive hybridization (“panhandle”) variant of PCR [38, 39]. The idea was to run PCR with primers with identical sequences on the 5′-ends that produce molecules capable of annealing with themselves and create pan-like structures. The shorter the molecule, the more probable it is that it forms pan-like structures and skips the ongoing PCR cycle. This gives longer amplicons a competitive advantage over primer-dimers and short non-specific PCR products. To achieve the effect, three primers needed to be included in the PCR mixture. They were a pair of primers with a specific 3′-part and “panhandle” 5′-tails that could be primed with the common “panhandle” primer and the “panhandle” primer itself. The “panhandle” primer has significantly higher annealing temperature, making it possible to employ a temperature switch toward “panhandle” annealing away from the specific primers annealing during the run of the PCR programme. We made use of this method in a long bisulfite PCR context and found that it allowed achieving much more stable PCR products than conventional bisulfite PCR (Fig. 1b). The concentration of specific primers in the reaction and the annealing temperature of the “panhandle” primer seems to be the key parameters for the optimisation (Additional file 1: Figure S1). The original barcoding strategy for the SMRT-BS [26] does not work well with PCR products with identical sequences on its ends. To address this, we used barcoding with ligation of Y-adapters [40] (Fig. 1c).

**Fig. 2:**
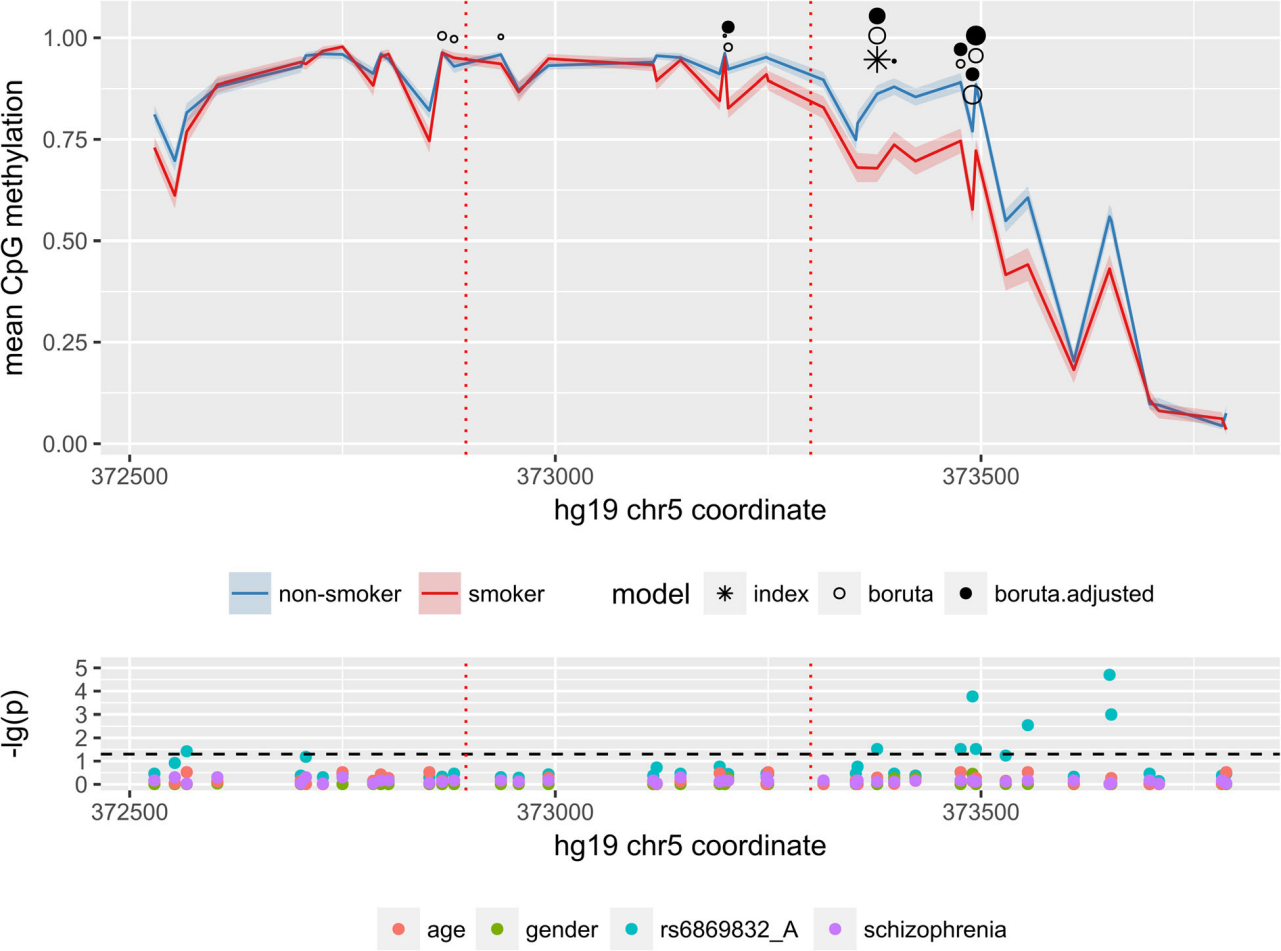
Methylation and ASM effects in the AHRR amplicon. *Top panel.* Mean methylation signal is shown in red (smokers) and blue (non-smokers) curves. Shaded areas of respective colour represent standard error. The symbols above the curves signify the reference CpG cg05575921 (star symbol) or important CpGs, selected by the Boruta algorithm (circles). Empty circles relate to the “boruta” model and black to the “boruta.adjusted” model. The size of a circle corresponds to the Boruta importance parameter. *Bottom panel.* Negative log_10_-transformed p-levels of “two-tailed” *t* test of different covariates (Benjamini-Hochberg adjusted) for the individual CpGs are portrayed. The p-levels are shown on the same genomic scale as in the top panel. Vertical dotted red lines on both panels indicate the location of the CpG-SNPs

We employed this improved “panhandle” SMRT-BS with a set of six smoking-related targets, which had already been established in a number of EWAS (Table 1) on DNA samples from the peripheral blood of 83 schizophrenia patients (27 smokers, 56 non-smokers) and 71 healthy controls (23 smokers, 48 non-smokers). After the sequencing, we were able to obtain enough data for five of them for analysis—AHRR, ALPPL2, IER3, GNG12 and GFI1 (named here in accord with a gene closest to the reference CpG). The absolute methylation signals for the reference CpGs (Additional file 1: Figure S2) and regional methylation profiles (Fig. 2 and Additional file 1: Figures. S3–6) were in agreement with other reports (Additional file 1: Figure S8).

**Fig. 3:**
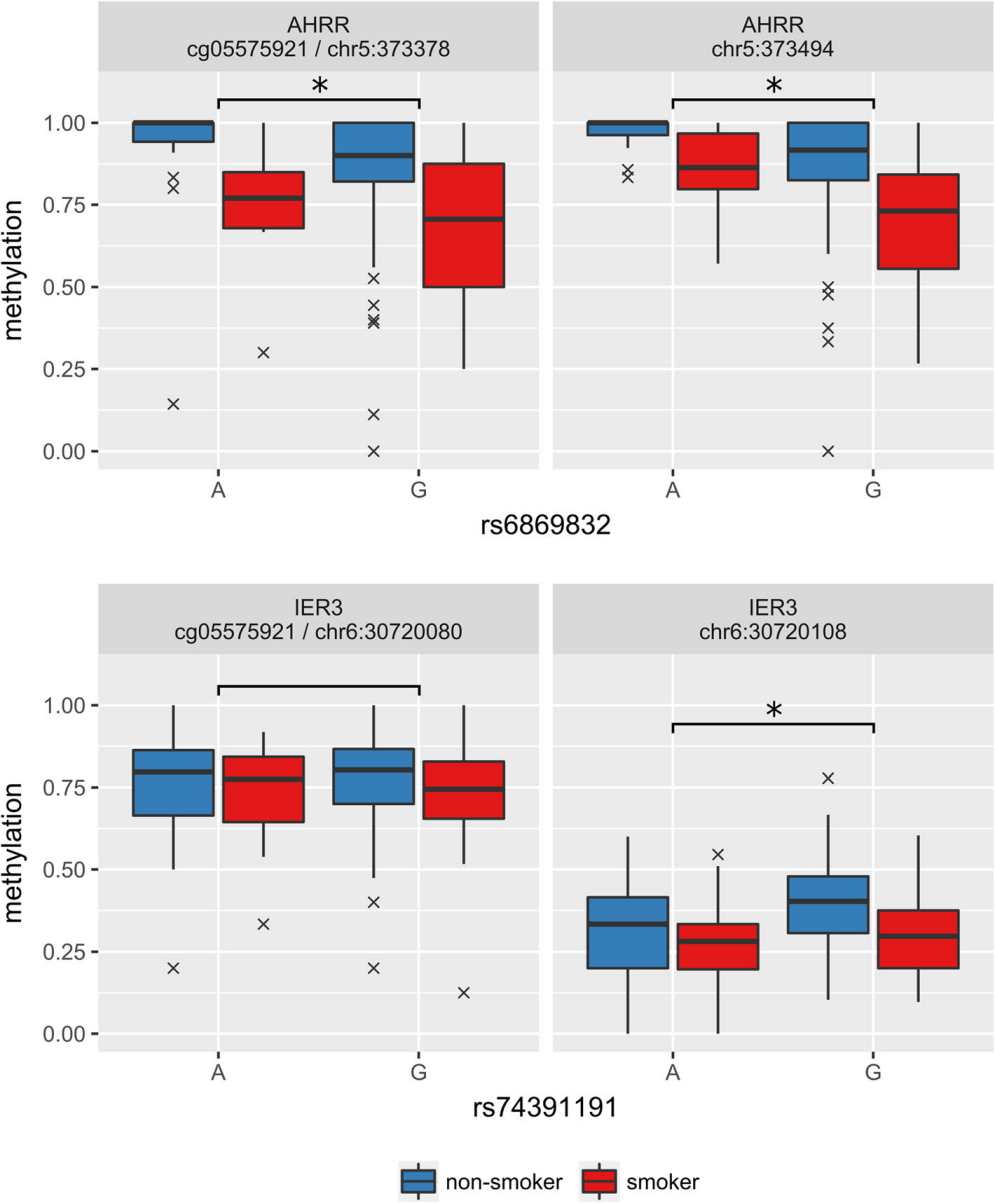
Selected ASM effects in the AHRR and IER3 target regions. Boxplots for methylation level are shown, boxplot whiskers represent the 25th and 75th percentiles. On the left are the reference CpGs, and on the right are the most important CpGs, selected by the “boruta.adjusted” model. The stars above the brackets denote significant ASM effect (*p <* 0.05, “two-tailed” *t* test)

To evaluate genetic-methylation interactions, we first divided methylation data according to identified haplotypes for each target and then adjusted them for age, gender, schizophrenia and local genetic factors. We found that ASM effects were present for the AHRR and IER3 targets (with minimum p-levels: *p* = 1.99E−05 for the AHRR target, CpG at chr5:373651 and *p* = 0.0098 for the IER3 target, CpG at chr6:30719450, “two-tailed” *t* test, Benjamini-Hochberg adjusted; Fig. 3 and Additional file 1: Figure S4). We found gender-specific methylation effects at a number of CpG sites within the ALPPL2 target (with minimum *p* = 0.037 for CpG at chr1:233284152; Additional file 1: Figure S3). Of note, methylation in the same region was already found to be the most affected by gender in EWAS [28]. No significant effects were found for either age or schizophrenia. The CpGs with significant effects are listed in Additional file 1: Table S4.

**Fig. 4:**
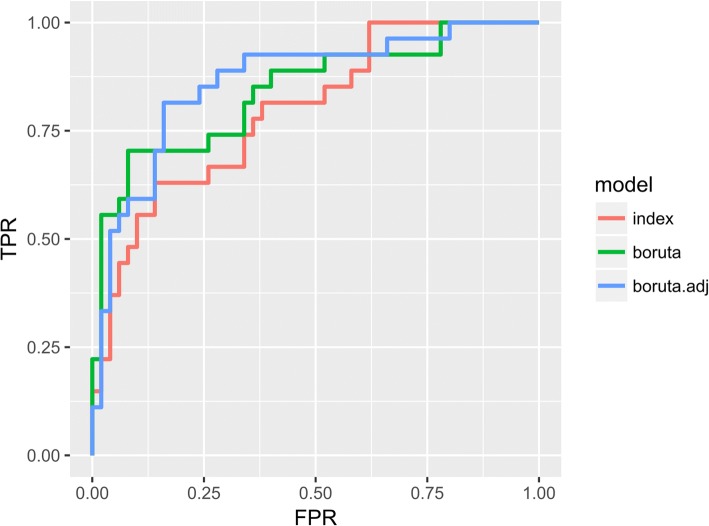
ROC plot for smoking prediction. The ROC curves for each of model based on combined five targets (“index”, “boruta” and “boruta.adjusted”) with true positive rate (TPR) plotted against false positive rate (FPR)

The ability to predict smoking status by methylation in those five targets was checked with three logistic regression models. First, the “index” model was the default model based on five CpGs identified in EWAS, one per target. The second model (“boruta”) was based on CpGs which were selected for each target by Boruta, a random forest-based feature selection algorithm [37]. The third model was the same as the second, but the data were additionally adjusted for genetic variations. The ROC curves for these models are presented in Fig. 4. We observed an improvement in the performance of the “boruta.adjusted” model (AUC = 0.861) over the “boruta” model (AUC = 0.836) and the “index” model (AUC = 0.796).

## Discussion

Here we describe an improved SMRT-BS method (“panhandle” SMRT-BS). The stable multiplex ability is the major improvement of the method. The most molecular events in the “panhandle” bisulfite PCR take place with the same “panhandle” primer, and undesirable products of PCR are suppressed. Multiplex bisulfite PCR followed by Pacbio sequencing (easily accessible via outsourcing) could be advantageous in clinical practice, for two important reasons. First, it presents a cheaper alternative to methylation arrays and could be easily implemented because most laboratories already have the necessary equipment, and secondly, it is potentially more precise because of multiple informative CpGs per target and explicit ASM effects.

In this work, we searched for important CpGs around major signals of EWAS of smoking. Only the strongest smoking EWAS signal (the AHRR target) coincided with CpGs detected by Boruta. When genetic variations were considered, another CpG (hg19 chr5:373494) was identified as the most relevant smoking predictor. Importantly, the same CpG was identified in a recent whole-genome bisulfite sequencing (WGBS) study [41]. The minor allele frequency of rs6869832 and other linked polymorphisms around the AHRR region is low in the Japanese population. This is probably the reason why methylation of this CpG was identified as the most affected by smoking regardless of genetic information. Therefore, we have shown that there is a strong chance of finding a better predictive CpG nearby an EWAS signal than the signal itself. This could potentially help formulate or refine hypotheses surrounding biological mechanisms behind the association. For example, in our work, the most “important” CpG in the GNG12 region was one particular CpG (hg19 chr1:68299400) located inside the EGR1 transcription factor binding site near the promoter of the *GNG12* gene, suggesting a link between smoking and EGR1-dependent regulation of the *GNG12*.

The long reads are especially useful for the study of the ASM effects. To assess for ASM, one must obtain both allelic and methylation signals on the same read. According to our estimates, the median distance between a polymorphism with minor allele frequency is more than 5% and a methylation probe from the Illumina 450K array is roughly 200 nucleotides for different populations (Additional file 1: Figure S7, top row), meaning that the every second CpG-polymorphism pair lies beyond the standard library size in methylation experiments. For studies of haplotype-specific methylation, this distance is expected to be almost three times greater (Additional file 1: Figure S7, bottom row). Additionally, thymines in C>T SNPs on the plus strand of bisulfite-converted DNA and guanines in G>A SNPs on the minus strand (apparently, the most common types of genetic variation) are indistinguishable from converted cytosines. This further limits a variety of potentially useful genetic information; however, this problem could be alleviated if reads on the opposite strand are available.

Though the long reads could provide additional information for DNA methylation-based trait prediction, the price for this is increased sequencing costs (compared with conventional Illumina sequencing), which account for most of the costs surrounding the described method. The sequencing of a nucleotide with the “third-generation” NGS platforms (Pacbio, Oxford Nanopore, and others) is yet more expensive than the same quality nucleotide with the Illumina platform (2018).

It is possible that phenotype-DNA methylation associations with strong ASM effects are less likely to be a result of EWAS because EWAS does not imply correction for genetic factors. Still, the ASM effects in our study were modest, but significant, probably because the ASM methodology is different and potentially more sensitive than the standard methylation quantitative trait loci (mQTL) analysis [22]. For example, Philibert et al. [42], in contrast to our study, did not detect a significant interaction between rs6869832 and methylation of the “index” CpG in the AHRR region.

The most similar approach, which allows obtaining methylation signal from multiple targeted genome locations, is the bisulfite padlock probe method [43]. The design of the probes is supposed to ensure specific binding, but nonspecific binding remains problematic [43]. The characteristic feature of the method is that the design of the probes is automated to select the most effective probes with a machine-learning algorithm. The selection is based on the data of preceding experiments, which helps discard undesirable probes including those prone to non-specific probe annealing [44]. It is difficult to compare padlock probes with SMRT-BS directly because the padlock probe method is aimed at Illumina sequencing of short stretches of the converted DNA. To the best of our knowledge, nobody has attempted to use padlock probes with increased length of targets, but it was established that the length of target sequences was highly correlated with capture efficiency of the target [45].

The yet-to-be-determined characteristic of “panhandle” SMRT-BS is the uniformity of methylation signal through the tested targets. In this work, we sequenced six targets and obtained a similar number of reads for all of the targets except for the CACNA1D. At this point, it is difficult to say why this happened to this particular target. As for the padlock probe design, more experience with massive multiplex experiments is necessary to elaborate the most effective primer design strategies. From a practical point of view, one could design two primer pairs on each of the strands of the target to avoid PCR failure as they stop being complementary after bisulfite conversion and do not interfere with each other.

Bisulfite PCR-based methods often suffer from PCR bias, a phenomenon when the amplification rate of an amplicon depends on its methylation status [46]. This leads to a skewed measure of methylation signal. Methylation profiles obtained in this work are similar to published WGBS profiles (Additional file 1: Figure S8). In fact, the long bisulfite PCR could be advantageous because longer amplicons have a more stable CpG/(CpG+CpH) ratio, making methylation signal differences within and between targets more relaxed.

The accurate measurement of methylation signal depends on how many reads survive filtering of raw data. The suggested approach could be improved to obtain more meaningful reads from the raw data. First, the 10-bp-long barcode sequence seems to be a suboptimal choice, especially if reads with lower quality scores are included in the analysis. The barcoding bias, though, is unavoidable because of the inefficient nature of the bisulfite PCR and could possibly be reduced with another barcoding strategy [47]. For example, the simple PCR-based solution could be to add a sequence for step-out PCR with barcoded sequences between panhandles and a specific sequence inside the primer sequence. Finally, we had to rely on a potentially too conservative de-duplication strategy to ensure that data contain no clonal artefacts. The ultimate solution is to incorporate the unique molecular identifiers (UMI) strategy [48] into the bisulfite PCR step, which could be easily implemented with, for example, the NOPE approach [49].

When building the prediction models, we assumed that genetic factors and smoking affected methylation independently. This seems to be a reasonable default assumption for EWAS signals, in particular, for the smoking-related regions, used in this study. However, it could be interesting to search for situations where this assumption does not hold and the ASM effects depend on a studied phenotype. The tempting opportunity is to explore this on a genomic scale with massive multiplex SMRT-BS.

In this work, we described a method, which could be utilised for creation of epigenetic clinical tests based on results of various EWAS. The approach allows for multiplex bisulfite PCR amplification of multiple targets followed by sequencing for evaluation of methylation signal around those targets with a single-based resolution. The use of long reads helps to correct for local polymorphisms, which could improve the accuracy of the test. The latter could be essential for a diagnosis of traits with complex gene-environment interactions, such as most of the common heritable diseases. We demonstrated how “panhandle” SMRT-BS works with the number of smoking-related targets. Smoking was chosen as a test phenotype for the method primarily because of a magnitude of smoking-related DNA methylation effects. The obtained results still may be useful by themselves as a guide for the creation of an objective biomarker for smoking exposure as people tend to under-report their smoking habits, for example, during routine check-ups [50, 51]. However, the clinical setting with more robust pack-years measure is required to develop such a test, which could be useful, for instance, for lung cancer prevention [52] or second-hand smoking evaluation [53].

We chose to validate our approach in a sample of schizophrenia patients and mentally healthy people. Based on research on smoking among psychiatry patients, we entertained the possibility that DNA methylation profiles of smoking were different in schizophrenia patients, which, if true, could be a diagnostic criterion for the disease itself. It is a well-established fact that schizophrenia patients tend to smoke more than mentally healthy people [54]. This suggests a link between the disease and smoking. The increased prevalence of smoking among patients is often perceived as self-medication [55, 56], but the issue remains controversial [57, 58]. It seems not accidental that the variability in the genes of cholinergic nicotinic receptors is a constant theme of genetic studies of schizophrenia [59–62]. This could be interpreted as if schizophrenia and smoking predisposition share the same biological background [56]. Though we were able to find already reported gender-specific differences in the methylation profile of the ALPPL2 target, we did not detect any significant difference in any of selected targets in relation to schizophrenia, suggesting that at least for these genomic sites, smoking methylation signatures were independent of a schizophrenia diagnosis.

## Conclusions

In this paper, we describe the “panhandle” modification of Yang et al.’s SMRT-BS method [27], which allows for more robust multiplex PCR of bisulfite-converted DNA. We applied this method to discriminate whole-blood DNA samples according to smoking exposure. We found that allele-specific information could improve the prediction accuracy of DNA methylation-based prediction models. In summary, the “panhandle” SMRT-BS method seems to be one of the plausible alternatives for studying targeted allele-specific methylation.

## Additional file

Additional file 1: **Table S1.** Oligonucleotide primers utilised in bisulfite PCR. **Table S2.** The sequences of oligonucleotides with molecular barcodes. **Table S3.** Polymorphisms used for the ASM analysis. **Table S4.** Significant methylation-covariate interactions. **Table S5.** Post-sequencing data preparation statistics. Supplementary Note 1. Computer simulations for the data in Fig. 1a. Supplementary Note 2. De-duplication procedure. **Figure S1.** Optimisation of the “panhandle” bisulfite multiplex PCR with the targets, used in the study. **Figure S2.** Boxplots of methylation signal in the index CpGs in smokers and non-smokers in comparison with published data. Figure S3. Methylation profiles of the ALPPL2 amplicon. **Figure S4.** Methylation profiles of the IER3 amplicon. **Figure S5.** Methylation profiles of the GNG12 amplicon. **Figure S6.** Methylation profiles of the GFI1 amplicon. **Figure S7.** Histogram of the closest nucleotide distances between SNPs and random 10,000 CpG probes from the Illumina 450K chip. Figure S8. Comparison of the obtained methylation profiles with published WGBS data. **Figure S9.** Scheme of the de-duplication strategy. (PDF 1730 kb)

## Abbreviations

450K: Illumina Infinium HumanMethylation450 BeadChip
ASM: Allele-specific methylation
AUC: Area under the curve
CGI: CpG island
EWAS: Epigenome-wide association study
GWAS: Genome-wide association study
mQTL: Methylation quantitative trait loci
NGS: Next-generation sequencing
PCR: Polymerase chain reaction
ROC: Receiver operating characteristic
SMRT-BS: Single-molecule real-time bisulfite sequencing
SNP: Single nucleotide polymorphism
WGBS: Whole-genome bisulfite sequencing
