# Correction to: Prediction of smoking by multiplex bisulfite PCR with long amplicons considering allele-specific effects on DNA methylation

**DOI:** 10.1186/s13148-018-0577-x

**Published:** 2018-12-06

**Authors:** Nikolay Kondratyev, Arkady Golov, Margarita Alfimova, Tatiana Lezheiko, Vera Golimbet

**Affiliations:** Clinical Genetics Laboratory, Mental Health Research Center, Moscow, Russia

## Correction

Upon publication of the original article [1], it was noticed that the Figure captions of Figs. 2 and 3 were incorrectly given. The correct Figure captions are given below.

Caption for Figure 2

Methylation and ASM effects in the AHRR amplicon.

Top panel. Mean methylation signal is shown in red (smokers) and blue (non-smokers) curves. Shaded areas of respective colour represent standard error. The symbols above the curves sygnify the reference CpG cg05575921 (star symbol) or important CpGs, selected by the Boruta algorithm (circles). Empty circles relate to the “boruta” model and black to the “boruta.adjusted” model. The size of a circle corresponds to the Boruta importance parameter.

Bottom panel. Negative log10-transformed p-levels of “two-tailed” t-test of different covariates (Benjamini-Hochberg adjusted) for the individual CpGs are portrayed. The p-levels are shown on the same genomic scale as in the top panel. Vertical dotted red lines on both panels indicate the location of the CpG-SNPs.

Caption for Figure 3

Selected ASM effects in the AHRR and IER3 target regions. Boxplots for methylation level are shown, boxplot whiskers represent the 25th and 75th percentiles. On the left are the reference CpGs, and on the right are the most important CpGs, selected by the “boruta.adjusted” model. The stars above the brackets denote significant ASM effect (*p* < 0.05, “two-tailed” t-test).

The equation in the Methods section was missing, the sentence ‘The methylation rate for each of the CpGs was logit-transformed according to the equation.’ should read as ‘The methylation rate for each of the CpGs was logit-transformed according to the equation: M=log(m’/1-m’); m’=(m(n-1)+0.5)/n, where m is raw methylation rate, n is the sample size.’ The original article has also been updated.
