# Supplementary material for: Prediction of smoking by multiplex bisulfite PCR with long amplicons considering allele-specific effects on DNA methylation
Source: Clin Epigenetics. 2018 Oct 23;10:130. doi: 10.1186/s13148-018-0565-1 (PMC6199807; doi:10.1186/s13148-018-0565-1)
Supplement: Supplementary file 1 — Table S1. Oligonucleotide primers utilised in bisulfite PCR. Table S2. The sequences of oligonucleotides with molecular barcodes. Table S3. Polymorphisms used for the ASM analysis. Table S4. Significant methylation-covariate interactions. Table S5. Post-sequencing data preparation statistics. Supplementary Note 1. Computer simulations for the data in Fig. 1a. Supplementary Note 2. De-duplication procedure. Figure S1. Optimisation of the “panhandle” bisulfite multiplex PCR with the targets, used in the study. Figure S2. Boxplots of methylation signal in the index CpGs in smokers and non-smokers in comparison with published data. Figure S3. Methylation profiles of the ALPPL2 amplicon. Figure S4. Methylation profiles of the IER3 amplicon. Figure S5. Methylation profiles of the GNG12 amplicon. Figure S6. Methylation profiles of the GFI1 amplicon. Figure S7. Histogram of the closest nucleotide distances between SNPs and random 10,000 CpG probes from the Illumina 450K chip. Figure S8. Comparison of the obtained methylation profiles with published WGBS data. Figure S9. Scheme of the de-duplication strategy. (PDF 1730 kb) [file 13148_2018_565_MOESM1_ESM.pdf]

Table S1
Oligonucleotide primers utilised in bisulfite PCR.

**Primer name**	**Sequence, 5′⟶ 3′**
AHRR_for	GCAGTCGAACATGTAGCTGACTCAGGTCACTGTGTATTTTGGGATGGGTTATAGGA
AHRR_rev	GCAGTCGAACATGTAGCTGACTCAGGTCACTCCCTAAACCTAAAAATCCCACCTAA
ALPPL2_for	GCAGTCGAACATGTAGCTGACTCAGGTCACGGGAAGAGAGATGTAATGAAGGTTTT
ALPPL2_rev	GCAGTCGAACATGTAGCTGACTCAGGTCACCCTACTTTCCCTTTCTCTCCTCTTA
IER3_for	GCAGTCGAACATGTAGCTGACTCAGGTCACTTTTTAGAGGTATGGGGGTAGAGTG
IER3_rev	GCAGTCGAACATGTAGCTGACTCAGGTCACCTCCCCTTATATACAATTCCCCTTCT
GNG12_for	GCAGTCGAACATGTAGCTGACTCAGGTCACAGGGTTTATTTGTTTTTAGTTGGTGAAA
GNG12_rev	GCAGTCGAACATGTAGCTGACTCAGGTCACCAACAAAACACTCTTCCCTCCTTC
GFI1_for	GCAGTCGAACATGTAGCTGACTCAGGTCACGAAAGGGGTTTTTGGGTTTAAATTGA
GFI1_rev	GCAGTCGAACATGTAGCTGACTCAGGTCACCACCTACCTCCTCTTTACAAACAAC
CACNA1D_for	GCAGTCGAACATGTAGCTGACTCAGGTCACTTTTAGGTAGTATTGGGAAGTAGAGGA
CACNA1D_rev	GCAGTCGAACATGTAGCTGACTCAGGTCACTTCCTTTTCAATCTATCTCACATCCAA

Table S2
The sequences of oligonucleotides with molecular barcodes.

Name of oligonucleotide	DNA sequence, 5′→ 3′, (P denotes 5′-phosphate modification)	Set
pY2ag_b1	P-CGAGTAGTGTTCATCTGCAAGGCACACAGGGGATAGG	1
pY2ag_b2	P-CGAGTAGTGTTCCAGTGCAAGGCACACAGGGGATAGG	1
pY2ag_b3	P-CGAGTAGTGTTCGCTCTCAAGGCACACAGGGGATAGG	1
pY2ag_b4	P-CGAGTAGTGTTCCGAGTCAAGGCACACAGGGGATAGG	1
pY2ag_b5	P-CGAGTAGTGTTCTAGCTCAAGGCACACAGGGGATAGG	1
pY2ag_b6	P-CGAGTAGTGTTCCATCGCAAGGCACACAGGGGATAGG	1
pY2ag_b7	P-CGAGTAGTGTTCAGCATCAAGGCACACAGGGGATAGG	1
pY2ag_b8	P-CGAGTAGTGTTCCTGCACAAGGCACACAGGGGATAGG	1
Y1ag_b9	CCATCTCATCCCTGCGTGTCTGTCTCTACACTACTCG	2
Y1ag_b10	CCATCTCATCCCTGCGTGTCCACGACTACACTACTCG	2
Y1ag_b11	CCATCTCATCCCTGCGTGTCTGAGACTACACTACTCG	2
Y1ag_b12	CCATCTCATCCCTGCGTGTCGACGTCTACACTACTCG	2
Y1ag_b13	CCATCTCATCCCTGCGTGTCTGTAGCTACACTACTCG	2
Y1ag_b14	CCATCTCATCCCTGCGTGTCACATGCTACACTACTCG	2
Y1ag_b15	CCATCTCATCCCTGCGTGTCCTAGTCTACACTACTCG	2
Y1ag_b16	CCATCTCATCCCTGCGTGTCGTGATCTACACTACTCG	2
Y1ag_b17	CCATCTCATCCCTGCGTGTCGTGCGCTACACTACTCG	2
Y1ag_b18	CCATCTCATCCCTGCGTGTCCTATCCTACACTACTCG	2
Y1ag_b19	CCATCTCATCCCTGCGTGTCGCTGACTACACTACTCG	2
Y1ag_b20	CCATCTCATCCCTGCGTGTCATACTCTACACTACTCG	2a*
Y1ag_b21	CCATCTCATCCCTGCGTGTCTCGCTCTACACTACTCG	2b*

* — Y1ag_b20 and Y1ag_b21 oligonucleotides were used to distinguish the libraries.

Table S3
Polymorphisms used for the ASM analysis.

SNP	Target	Alleles	Chromosome	Genome coordinate (hg19)
rs6869832	*AHRR*	G/A	chr5	373300
rs2678520	*ALPPL2*	A/T	chr2	233284285
rs13017092	*ALPPL2*	C/T	chr2	233284814
rs74391191	*IER3*	G/A	chr6	30719588
rs11209163	*GNG12*	A/C	chr1	68299291
rs12136827	*GNG12*	C/T	chr1	68299331
rs6588283	*GNG12*	C/T	chr1	68299797
rs11448559	*GFI1*	indel	chr1	92947981

Table S4
Significant methylation-covariate interactions.

Target	Chromosome	Genome coordinate (hg19)	Covariate	p-level, “two-tailed” t-test, Benjamini-Hochberg adjusted
AHRR	chr5	372567	rs6869832	0.0377
AHRR	chr5	373378	rs6869832	0.0301
AHRR	chr5	373476	rs6869832	0.0301
AHRR	chr5	373490	rs6869832	1.7E-04
AHRR	chr5	373494	rs6869832	0.0301
AHRR	chr5	373555	rs6869832	0.0028
AHRR	chr5	373651	rs6869832	1.99E-05
AHRR	chr5	373653	rs6869832	0.0010
ALPPL2	chr1	233284152	gender	0.0368
ALPPL2	chr1	233284218	gender	0.0369
ALPPL2	chr1	233284675	gender	0.0391
ALPPL2	chr1	233284775	gender	0.0391
IER3	chr6	30719450	rs74391191	0.0098
IER3	chr6	30720108	rs74391191	0.0199
IER3	chr6	30720261	rs74391191	0.0445
IER3	chr6	30720491	rs74391191	0.0445

Table S5
Post sequencing data preparation statistics.

Target	Reads with both correct primer sequences	Reads with both correct barcode sequences	Mapped reads	Reads with less than 0.05 CpH methylation	Reads after de-duplica tion	Number of meaningful CpH
AHRR	4661	4166	3579	3482	3287	46
ALPPL2	8826	7906	7144	7100	3295	324
IER3	22499	20006	17825	16208	16185	356
GNG12	11065	9798	8467	8428	4232	280
GFI1	9382	8393	7208	7100	3212	323
CACNA1D	148	136	131	9	NA	NA

Supplementary Note 1
Computer simulations for the data in Figure 1A

Differences in PCR efficiency between conventional and bisulfite-converted DNA were tested in two realistic scenarios of the selection of primers for the unconverted (scenario A) and converted matrix (scenario B). The target was chosen to be a random letter inside 1000 randomly selected regions UCSC CGI +/- 4000 bp (by definition: CGI + shores + shelves) with a fraction of sequences within the RepeatMasker base of no more than 10%; in scenario B, a DNA strand was also randomized. The same set of targets was used for both scenarios A and B. Primer3 v2.3.7 software [1] with parameters Tm = 65±5°C and 1000 bp amplicon length (+/- 50bp) was used to select the primers. The minimum, desired, and maximum primer lengths for scenario A were set as 18/22/28, and for scenario B, 24/26/35, respectively. The rest of the parameters were set at default. In scenario B, all CpGs were excluded from the primer selection. The reported thermodynamic parameters, PRIMER_PAIR_MAX_COMPL_ANY_TH, and PRIMER_MAX_SELF_ANY_TH were utilised as an estimate of the tendency of primers to form primer-dimers, with themselves and among themselves, respectively. The nucleotide BLAST program [2] was applied to define the number of hits with the 3′-portion of an oligonucleotide (16 letters without mismatches). This value was used as an estimate for the primers to anneal to nonspecific sites in the unconverted genome for scenario A or converted for scenario B.

Supplementary Note 2
De-duplication procedure

The de-duplication data postprocessing used in this paper was based on bisulfite conversion artefacts at CpH sites. We removed all reads with any unconverted cytosine in a CpH context at the same position except that with the best base quality score. To compensate for the loss of specificity, we added an additional filtering procedure for the unconverted cytosines in the CpH context that are overrepresented in the data across individuals. The aim of this step is to identify unconverted cytosines which could potentially represent the true methylation signal.

The filtration procedure has the following steps (Figure S9):

I. First, we count, how many individuals, n_i, has at least one unconverted cytosine, i, for each CpH in a sequenced amplicon. Second, we estimate mean conversion rate for a given PCR product, c, and the set of probabilities, p_j, that for an individual, j, with k_j reads and a given c has at least one read with unconverted cytosine (modelled as Bernoulli trials). Note that p_j is expected to be overestimated, c is slightly underestimated and n_i overestimated because of certain reads could indeed be the clonal artefacts.

$$p_j = 1 - c^{k_j}$$

II. Next, we model probability, $P_i(n \geq n_i)$, to have at least n_i for a given CpH with Poisson binomial distribution with the set of estimated p_j with c.d.f. Φ for normal distribution (normal approximation method [3]):

$$P_i(n \geq n_i) = 1 - \Phi\left(\frac{n_i - 0.5 - \mu}{\sigma}\right) \text{ ; where } \mu = \sum_j p_j \text{ and } \sigma = \left[\sum_j p_j(1 - p_j)\right]^{0.5}$$

III. We mark reads that could be clonal artefacts and repeat the algorithm without them from step I with updated p_j, c and n_i. We proceed to de-duplication when the relative change in μ is less than 0.1%. Every CpH with the final $P_i(n \geq n_i) < 0.05$ was not employed for the de-duplication.

The procedure leaves plenty of CpH's to check for clonal artefacts (Table S5):

Note that genuine CpH methylation, while rare, is still a widespread phenomenon [4], especially prominent in neuronal cells [5]. Though we believe that the described de-duplication procedure is as best as possible for this type of data, a more robust option is to incorporate the unique molecular identifiers (UMI) strategy [6] into the bisulfite PCR step.

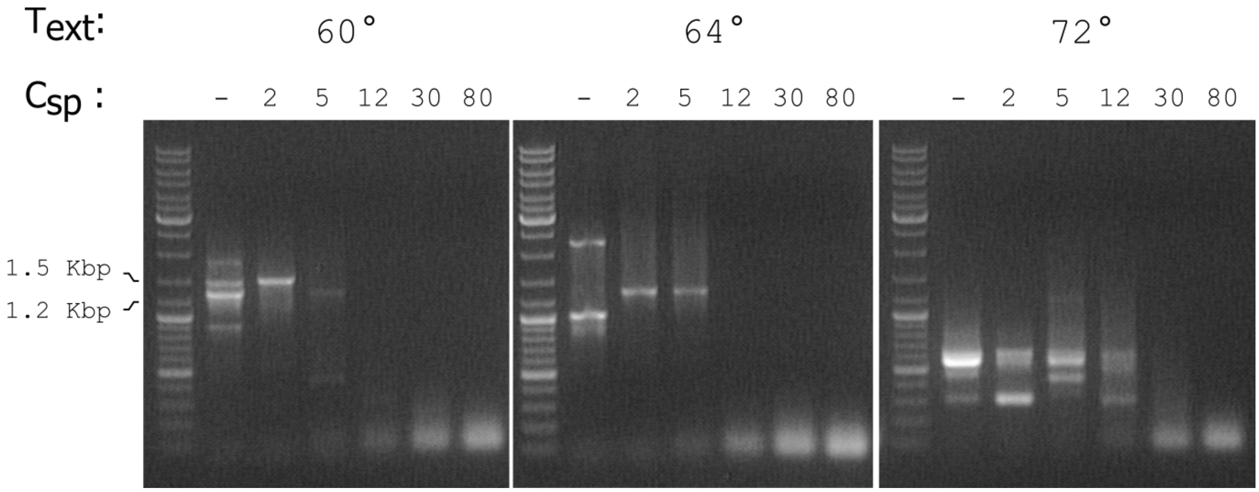

Figure S1
Optimisation of the "panhandle" bisulfite multiplex PCR with the targets, used in the study. T_{ext} denotes the temperature of the combined annealing/polymerisation stage of the "panhandle" bisulfite PCR. C_{sp} denotes the final nM concentration of each of the specific primer in the reaction.

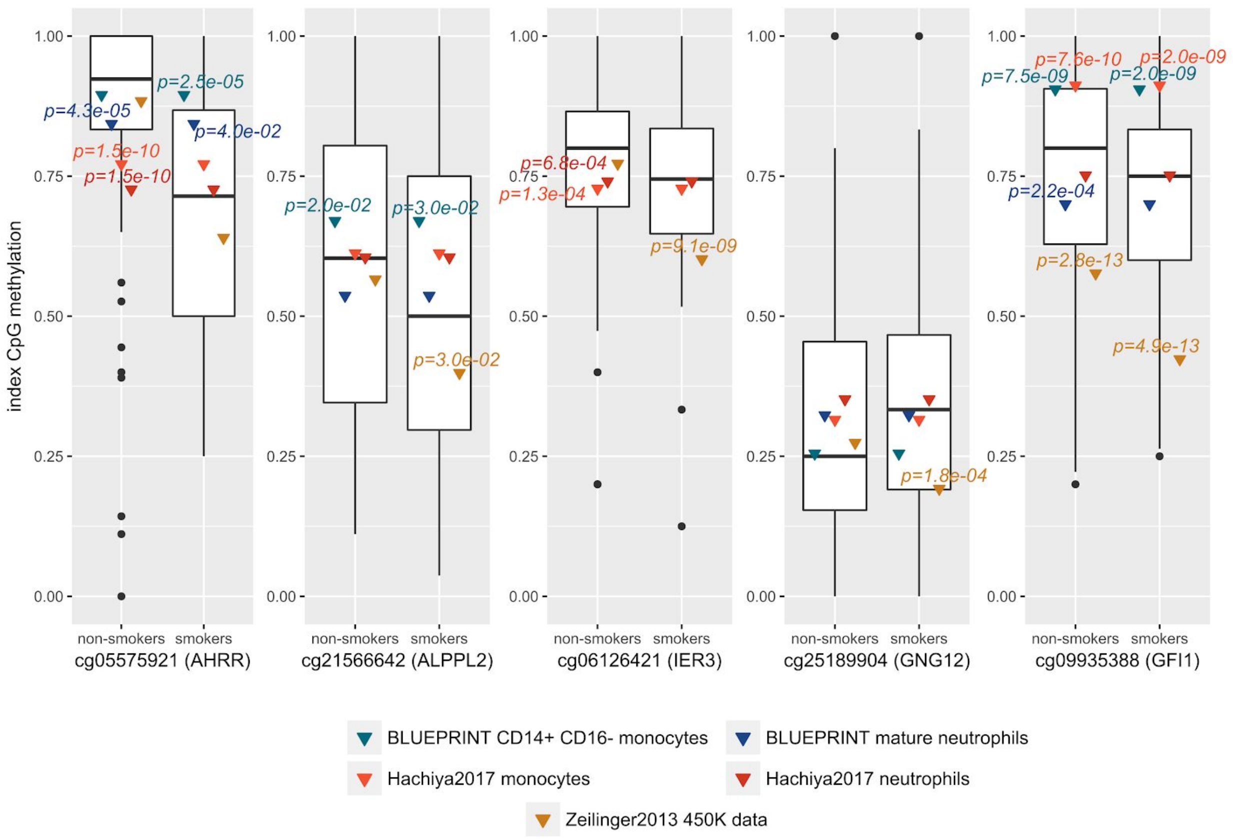

Figure S2

Boxplots of methylation signal in the index CpGs in smokers and non-smokers in comparison with published data. The methylation signal in the index CpGs from the three datasets are presented. Blue triangles signify mean values from BLUEPRINT consortium WGBS data [7], red triangles are the mean methylation signal from summary data from Hachiya et al WGBS [8]. BLUEPRINT and Hachiya WGBS data from two cell types from peripheral blood are presented: neutrophils (darker triangles) and CD14+/CD16- monocytes (lighter triangles). The BLUEPRINT data for IER3 region is not shown because the data is absent. Yellow triangles signify Zeilinger et al EWAS data [9]. Whenever published methylation signals differentiate from data in the paper ($p < 0.05$, two-sided one-sample sign test), the p-value is shown close to the data point.

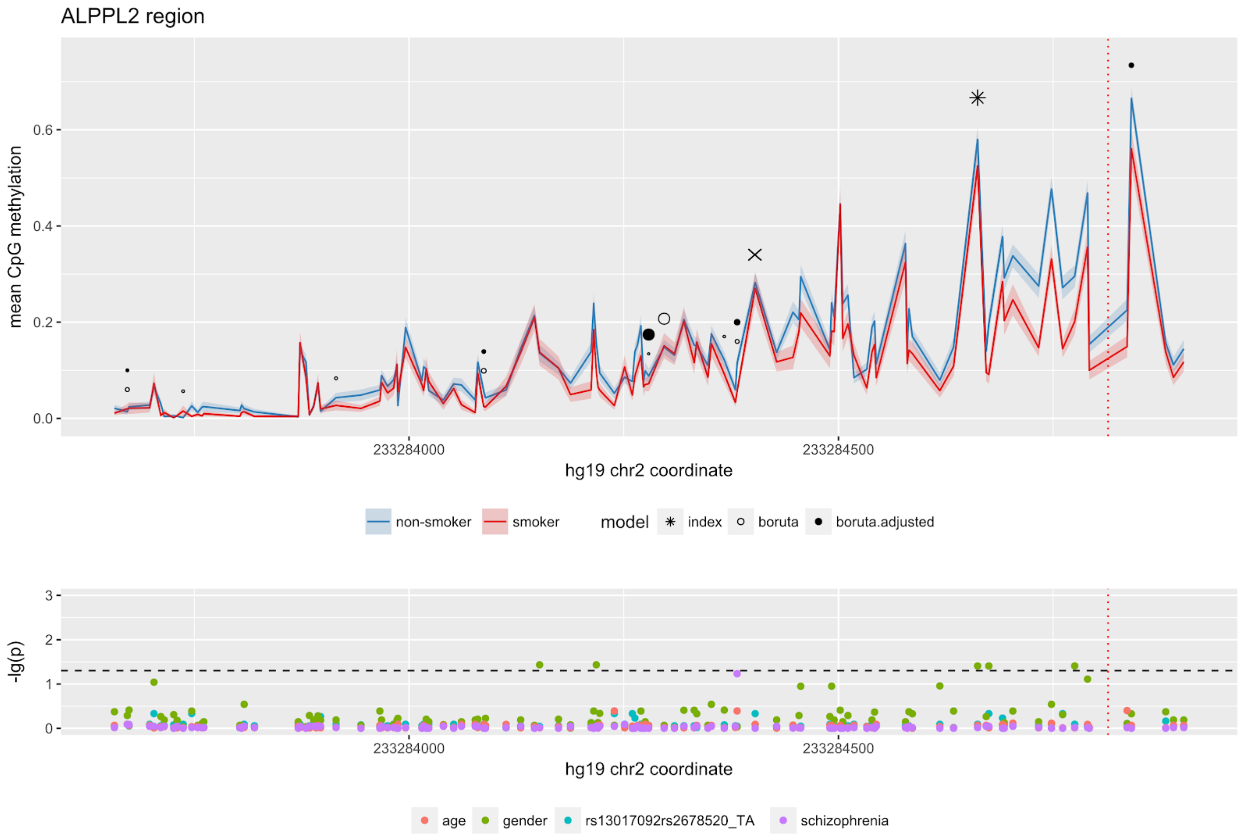

Figure S3

Methylation profiles of the ALPPL2 amplicon.

Top panel. Mean methylation signal is shown in red (smokers) and blue (non-smokers) curves. Shaded areas of respective colour represent standard error. The symbols above the curves signify either reference CpG cg21566642 (star symbol) or important CpGs, selected by the Boruta algorithm (circles). Empty circles relate to "boruta" model and black to "boruta.adjusted" model. Ex-mark signifies the position of another reference CpG cg05951221, usually reported being associated with smoking alongside with cg21566642.

Bottom panel. Negative log_{10}-transformed p-levels of "two-tailed" t-test of different covariates (Benjamini-Hochberg adjusted) for individual CpGs are portrayed. The p-levels are shown on the same genomic scale as in the top panel. Vertical dotted red lines on both panels indicate the location of the CpG-SNPs.

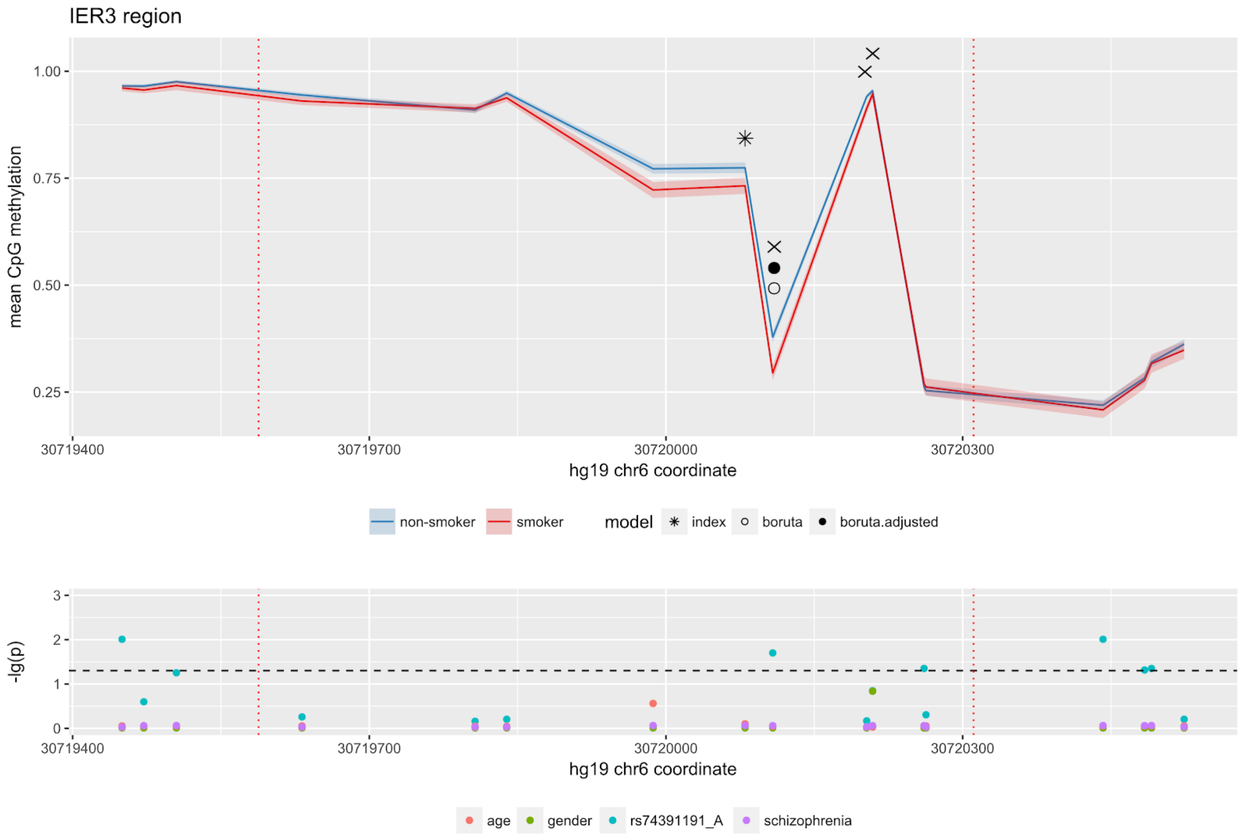

Figure S4

Methylation profiles of the IER3 amplicon.

Top panel. Mean methylation signal is shown in red (smokers) and blue (non-smokers) curves. Shaded areas of respective colour represent standard error. The symbols above the curves signify either reference CpG cg06126421 (star symbol) or important CpGs, selected by Boruta algorithm (circles). Empty circles relate to “boruta” model and black to “boruta.adjusted” model. Ex-marks signify positions of another reference CpGs, usually reported to be associated with smoking alongside with cg06126421: cg14753356, cg24859433, and cg15342087 (from left to right).

Bottom panel. Negative $\log_{10}$-transformed p-levels of "two-tailed" t-test of different covariates (Benjamini-Hochberg adjusted) for individual CpGs are portrayed. The p-levels are shown on the same genomic scale as in the top panel. Vertical dotted red lines on both panels indicate the location of the CpG-SNPs.

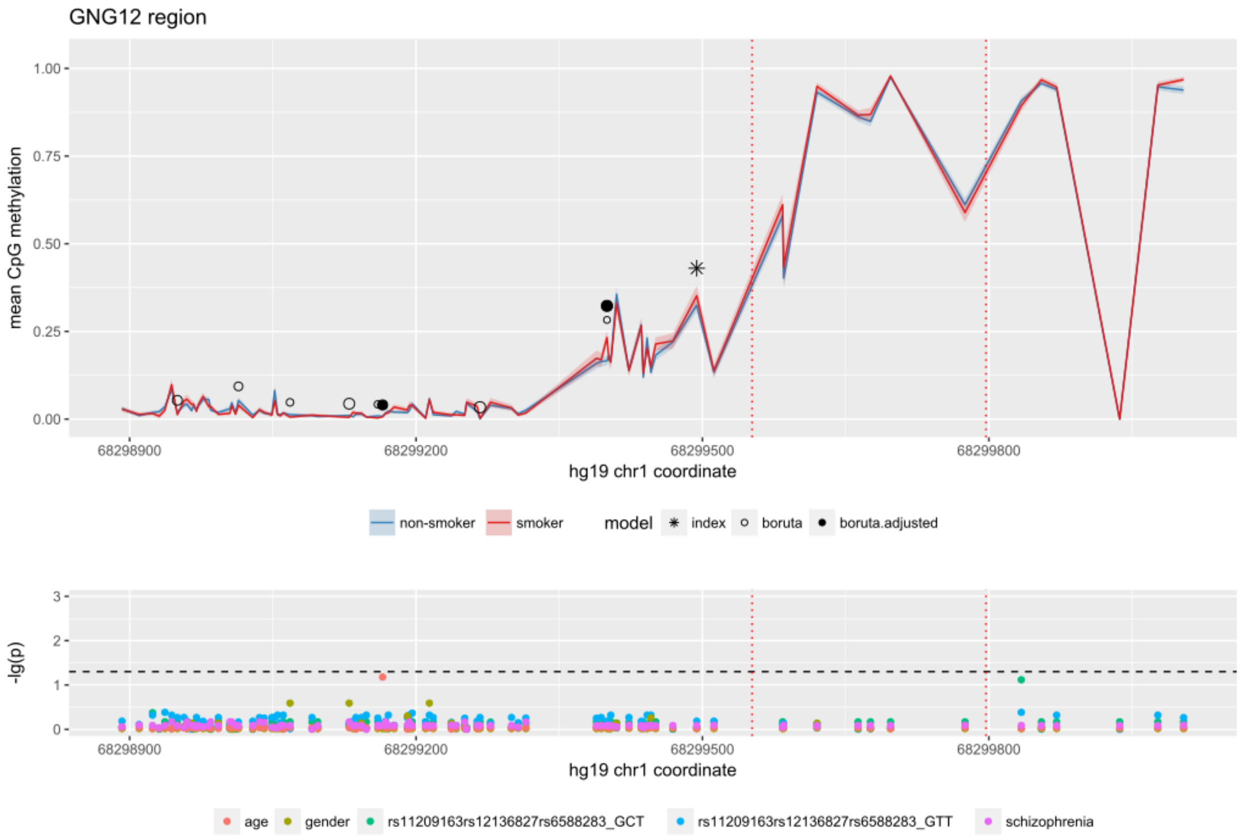

Figure S5

Methylation profiles of the GNG12 amplicon.

Top panel. Mean methylation signal is shown in red (smokers) and blue (non-smokers) curves. Shaded areas of respective colour represent standard error. The symbols above the curves signify either reference CpG cg25189904 (star symbol) or important CpGs, selected by Boruta algorithm (circles). Empty circles relate to “boruta” model and black to “boruta.adjusted” model.

Bottom panel. Negative log_{10}-transformed p-levels of "two-tailed" t-test of different covariates (Benjamini-Hochberg adjusted) for individual CpGs are shown. The p-levels are shown on the same genomic scale as in the top panel. Vertical dotted red lines on both panels indicate the location of CpG-SNPs.

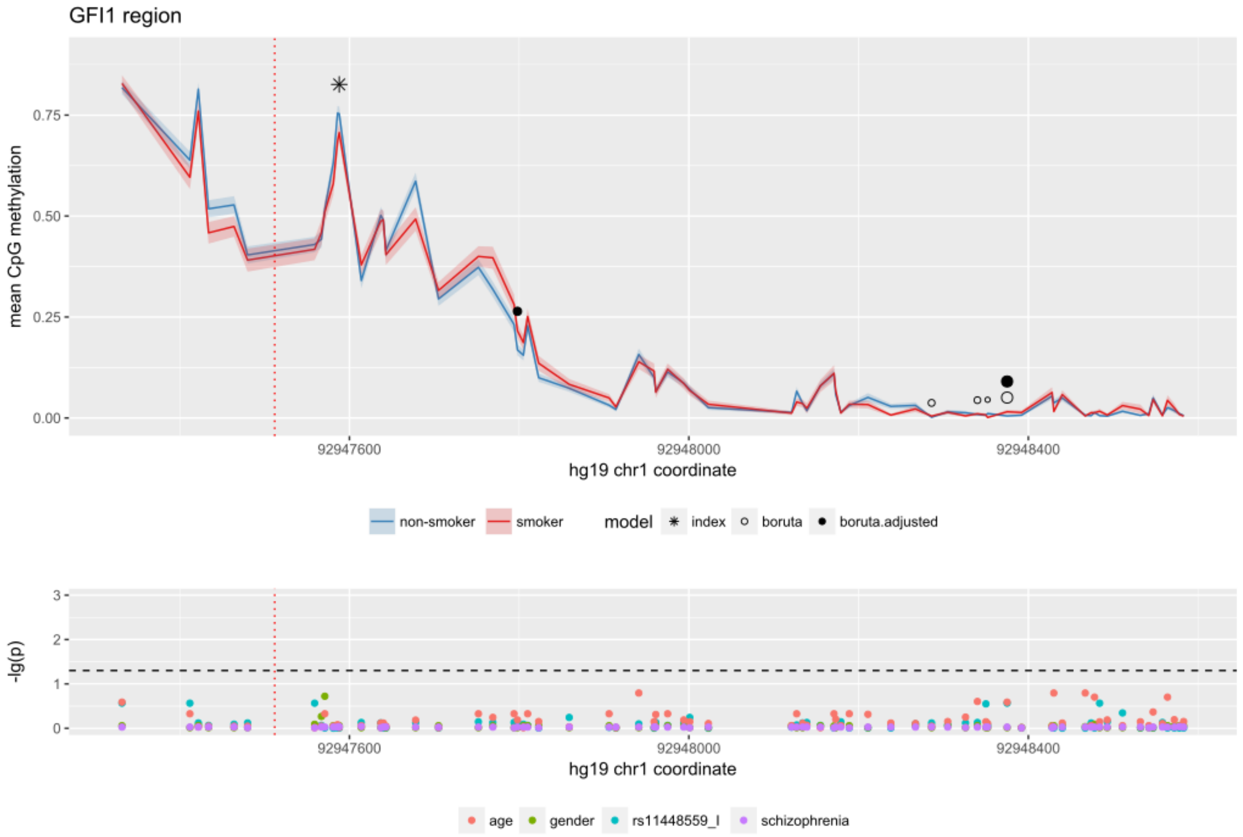

Figure S6

Methylation profiles of the GFI1 amplicon.

Top panel. Mean methylation signal is shown in red (smokers) and blue (non-smokers) curves. Shaded areas of respective colour represent standard error. The symbols above the curves signify either reference CpG cg09935388 (star symbol) or important CpGs, selected by Boruta algorithm (circles). Empty circles relate to “boruta” model and black to “boruta.adjusted” model.

Bottom panel. Negative log_{10}-transformed p-levels of "two-tailed" t-test of different covariates (Benjamini-Hochberg adjusted) for individual CpGs are shown. The p-levels are shown on the same genomic scale as in the top panel. Vertical dotted red lines on both panels indicate the location of the CpG-SNPs.

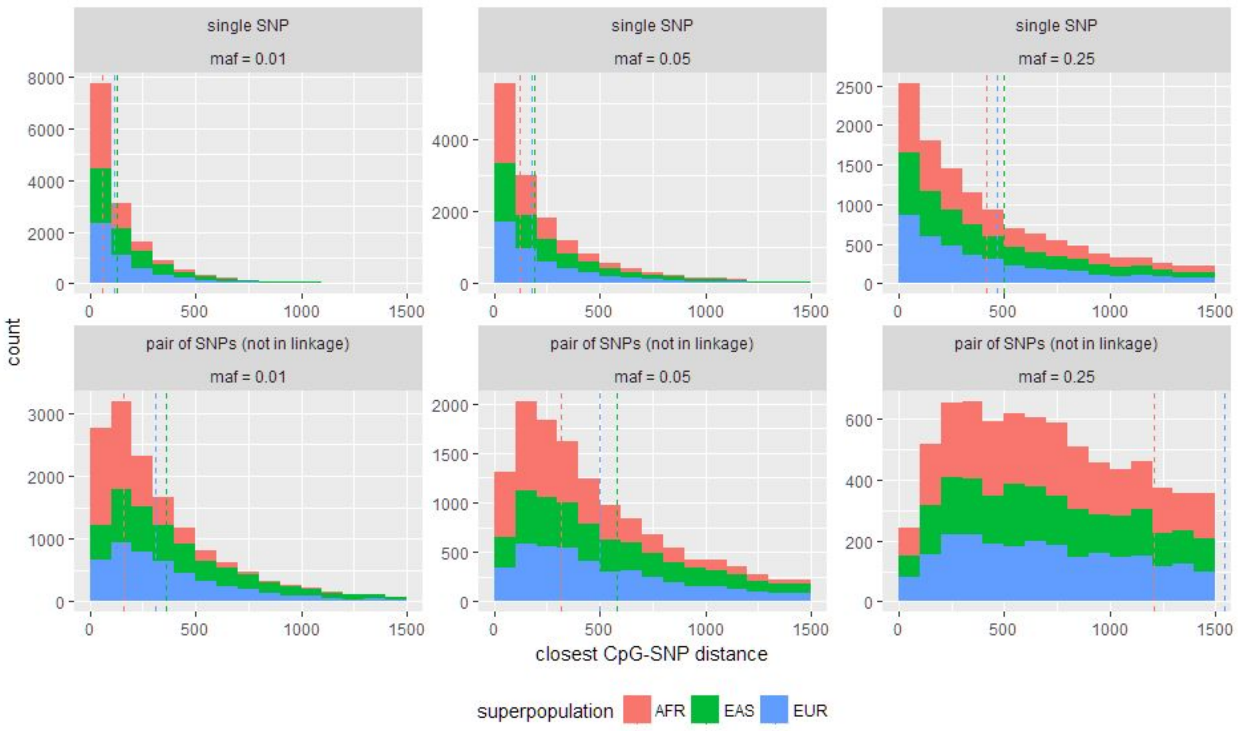

Figure S7

Histogram of the closest nucleotide distances between SNPs and random 10000 CpG probes from the Illumina 450K chip. The SNPs were selected to have a minor allele frequency (MAF) above the specified threshold in a given population (1%, 5% and 25%, in the vertical columns). The 1000 genomes Project (Phase 3) data were used [10]. The 1000g "superpopulations" AFR, EAS and EUR are colour-coded. The top row represents the histogram of distances between CpG and its closest SNP with MAF no less than the threshold in a given population. The bottom row represents the distance between the CpG and the farthest SNP in the closest pair of SNPs, which are not perfectly linked ($r^2 < 0.9$, computed with LDlink [11]) with each other. The vertical lines indicate the median distances.

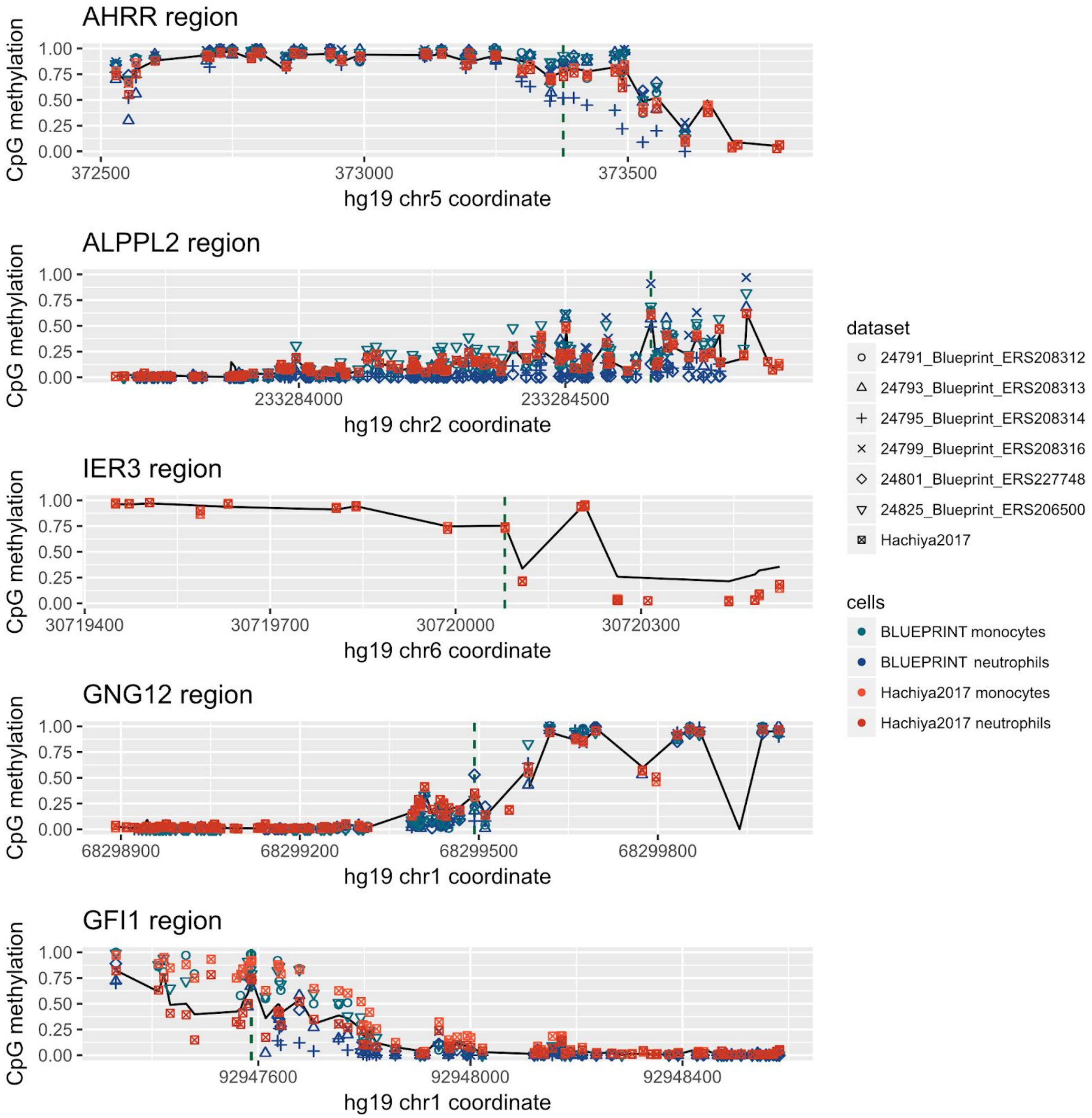

Figure S8

Comparison of the obtained methylation profiles with published WGBS data. Methylation profiles, obtained in this work, are compared with the whole-genome bisulfite sequence (WGBS) data of methylation in two cell types of peripheral blood (neutrophils and CD14+/CD16- monocytes), published by the BLUEPRINT consortium (blue symbols) [7] and Hachiya et al (red symbols) [8]. BLUEPRINT data for the IER3 target is not shown because the data could not be retrieved (the target resides inside the MHC cluster). Black lines represent average profiles of methylation across all individuals. The WGBS methylation profiles for BLUEPRINT data in the target regions were retrieved from corresponding bigWig files with the bwtool software [12]. Vertical green lines indicate positions of the index CpGs of smoking EWAS.

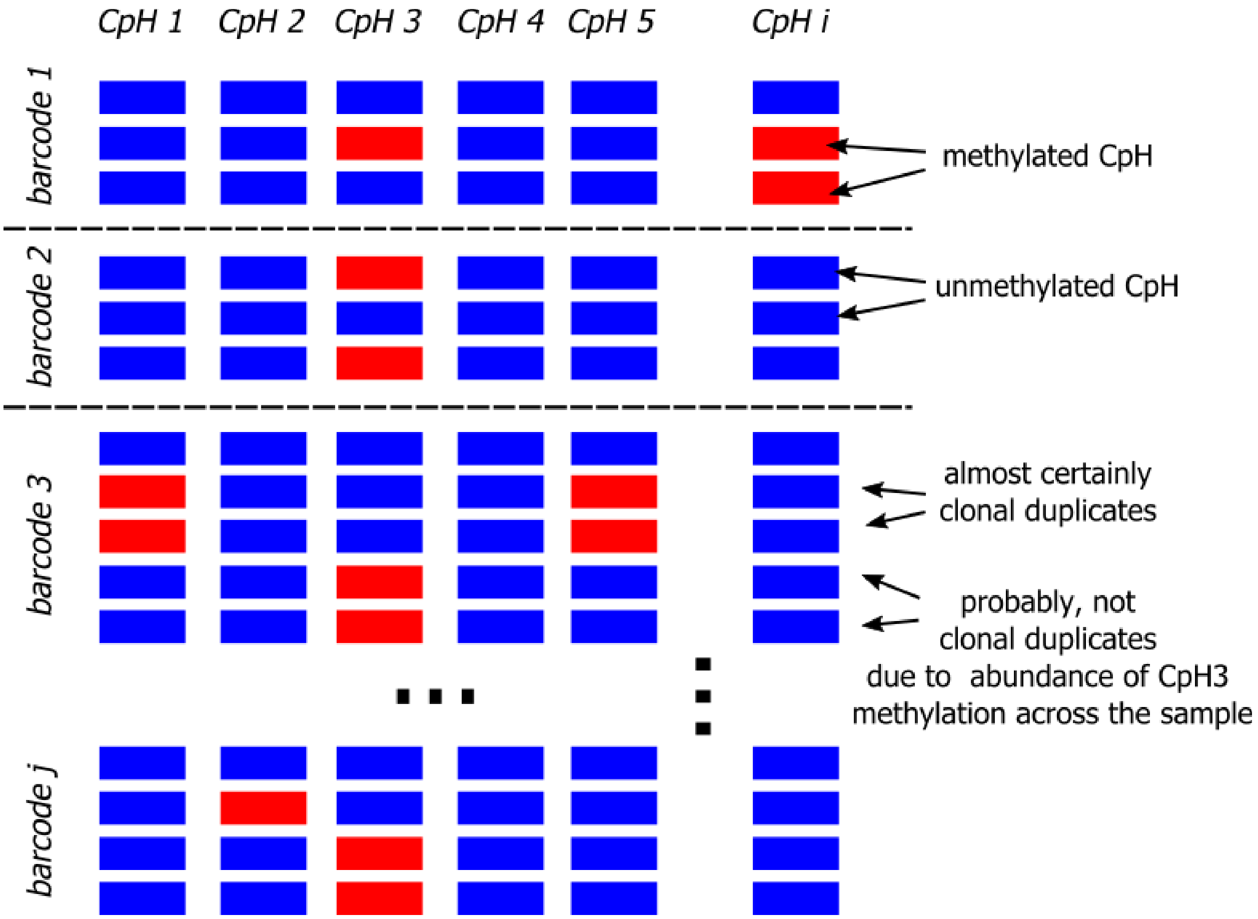

Figure S9
Scheme of the de-duplication strategy. The rows are the separate reads, grouped by barcodes. The columns indicate CpHs.

Additional file references

1. Untergasser A, Cutcutache I, Koressaar T, Ye J, Faircloth BC, Remm M, et al. Primer3--new capabilities and interfaces. Nucleic Acids Res. 2012;40:e115.

2. Altschul SF, Gish W, Miller W, Myers EW, Lipman DJ. Basic local alignment search tool. J Mol Biol. 1990;215:403–10.

3. Hong Y. On Computing the Distribution Function for the Poisson Binomial Distribution. Comput Stat Data Anal. Amsterdam, The Netherlands, The Netherlands: Elsevier Science Publishers B. V.; 2013;59:41–51.

4. Ziller MJ, Müller F, Liao J, Zhang Y, Gu H, Bock C, et al. Genomic distribution and inter-sample variation of non-CpG methylation across human cell types. PLoS Genet. 2011;7:e1002389.

5. Patil V, Ward RL, Hesson LB. The evidence for functional non-CpG methylation in mammalian cells. Epigenetics. 2014;9:823–8.

6. Smith T, Heger A, Sudbery I. UMI-tools: modeling sequencing errors in Unique Molecular Identifiers to improve quantification accuracy. Genome Res. 2017;27:491–9.

7. Adams D, Altucci L, Antonarakis SE, Ballesteros J, Beck S, Bird A, et al. BLUEPRINT to decode the epigenetic signature written in blood. Nat Biotechnol. 2012;30:224–6.

8. Hachiya T, Furukawa R, Shiwa Y, Ohmomo H, Ono K, Katsuoka F, et al. Genome-wide identification of inter-individually variable DNA methylation sites improves the efficacy of epigenetic association studies. NPJ Genom Med. 2017;2:11.

9. Zeilinger S, Kühnel B, Klopp N, Baurecht H, Kleinschmidt A, Gieger C, et al. Tobacco smoking leads to extensive genome-wide changes in DNA methylation. PLoS One. 2013;8:e63812.

10. 1000 Genomes Project Consortium, Auton A, Brooks LD, Durbin RM, Garrison EP, Kang HM, et al. A global reference for human genetic variation. Nature. 2015;526:68–74.

11. Machiela MJ, Chanock SJ. LDlink: a web-based application for exploring population-specific haplotype structure and linking correlated alleles of possible functional variants. Bioinformatics. 2015;31:3555–7.

12. Pohl A, Beato M. bwtool: a tool for bigWig files. Bioinformatics. 2014;30:1618–9.
